# Improvements in structural and optical properties of wafer-scale hexagonal boron nitride film by post-growth annealing

**DOI:** 10.1038/s41598-019-47093-9

**Published:** 2019-07-22

**Authors:** Seung Hee Lee, Hokyeong Jeong, Odongo Francis Ngome Okello, Shiyu Xiao, Seokho Moon, Dong Yeong Kim, Gi-Yeop Kim, Jen-Iu Lo, Yu-Chain Peng, Bing-Ming Cheng, Hideto Miyake, Si-Young Choi, Jong Kyu Kim

**Affiliations:** 1 Department of Materials Science and Engineering, Pohang University of Science and Technology (POSTECH), Pohang, 37673 Republic of Korea; 2 Graduate School of Regional Innovation Studies, Mie University, Tsu, 514-8507 Japan; 3 National Synchrotron Radiation Research Center, Hsinchu, 30076 Taiwan

**Keywords:** Two-dimensional materials, Structural properties

## Abstract

Remarkable improvements in both structural and optical properties of wafer-scale hexagonal boron nitride (h-BN) films grown by metal-organic chemical vapor deposition (MOCVD) enabled by high-temperature post-growth annealing is presented. The enhanced crystallinity and homogeneity of the MOCVD-grown h-BN films grown at 1050 °C is attributed to the solid-state atomic rearrangement during the thermal annealing at 1600 °C. In addition, the appearance of the photoluminescence by excitonic transitions as well as enlarged optical band gap were observed for the post-annealed h-BN films as direct consequences of the microstructural improvement. The post-growth annealing is a very promising strategy to overcome limited crystallinity of h-BN films grown by typical MOCVD systems while maintaining their advantage of multiple wafer scalability for practical applications towards two-dimensional electronics and optoelectronics.

## Introduction

Hexagonal boron nitride (h-BN) is a two-dimensional (2D) layered material based on *sp*^2^ covalent bonds between boron and nitrogen atoms^1,2^. It has gained a great deal of attention as a fundamental building block for 2D materials-based electronic and optoelectronic devices^3^ due to its atomically flat surface without dangling bonds, remarkable electronic properties such as wide energy bandgap (~6 eV)^4–7^, low dielectric constant^8,9^ and high dielectric strength^10,11^, and high chemical and thermal stability^12–14^. In addition, h-BN is a promising active material for deep ultraviolet (DUV) optoelectronics with a high emission efficiency originating from the strong light-matter interaction of 2D materials^4,6,15–17^. There are various strategies to obtain atomically-thin h-BN layers. Although mechanically exfoliated mono- or few- layer h-BN from bulk h-BN crystal synthesized under high pressure and high temperature^4^ exhibits excellent crystalline quality, there are significant difficulties in scalability and controllability over thickness and size, limiting its applications with practical 2D materials-based devices^18^. On the other hands, chemical vapor deposition (CVD) methods offer a promising opportunity in the growth of a large-area h-BN film with a high crystallinity^19–23^. However, multiple wafer-scale production of h-BN films, compatible with current micro-electronic technologies, remains significantly restricted due to limited quartz tube diameter and heating zone in conventional CVD systems.

Metal-organic chemical vapor deposition (MOCVD) method is a well-matured commercially-successful technology especially for multiple wafer-scale epitaxial growth of various compound semiconductors including gallium nitride. Recently, MOCVD method has been proposed for scalable and low-cost production of h-BN films. Layered h-BN film was successfully grown on a sapphire substrate by using MOCVD as a release layer for mechanical transfer of GaN-based devices^24^ and an active layer for DUV optoelectronics^11^. In addition, wafer-scale growth of h-BN films on sapphire substrates and their dependency on growth parameters such as growth temperature and pressure, types of carrier gases, flow rates of precursors, precursor ratio, and growth modes have been systematically investigated^25–29^. Among them, growth temperature plays a predominant role in determining the crystal quality of h-BN films. It was reported that the growth temperature greater than 1500 °C is required to obtain a highly crystalline film^28–30^, which is far beyond the capability of typical MOCVD systems less than ~1200 °C. High temperature post-growth annealing would be a promising way to enhance the crystallinity of the h-BN film grown at moderate temperature by solid phase epitaxial regrowth^31–33^, while maintaining the advantages offered by MOCVD.

In this work, we have demonstrated remarkable improvements in the crystallinity and the optical properties of the 2-inch wafer-scale h-BN film grown by MOCVD at 1050 °C by high-temperature (1500–1700 °C) post-growth annealing. The ordering of h-BN domains accompanied by atomic rearrangements after the post-growth annealing at 1600 °C in nitrogen (N_2_) ambient was observed by transmission electron microscopy (TEM) and near-edge X-ray absorption fine-structure (NEXAFS) analysis. Furthermore, it was found that the enhanced crystallinity by the post-growth annealing results in the appearance of the photoluminescence by excitonic transitions, as well as enlarged optical band gap in the h-BN. We believe that our results offer a promising and practical route for obtaining highly crystalline and multiple wafer-scale h-BN films for 2D electronic and optoelectronic devices.

## Results and Discussion

**Figure 1:**
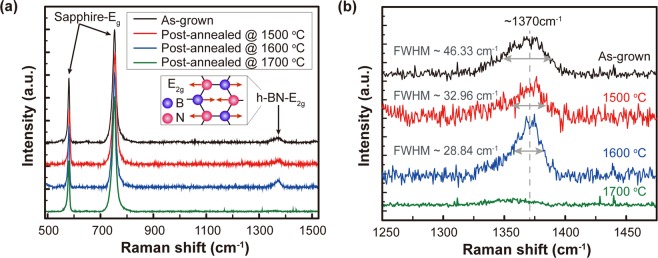
(**a**) Comparison of the full Raman spectra of the MOCVD-grown h-BN film on sapphire before and after the post-annealing from 1500 to 1700 °C in N_2_ ambient for 30 min. (**b**) The magnified view of the h-BN E_2g_ peaks at 1370 cm^−1^.

Figure 1 shows Raman spectra of the as-grown and the post-annealed MOCVD-grown few-layer h-BN films (~3.07 nm) on a 2-inch sapphire substrate at 1500, 1600, 1700 °C in N_2_ ambient for 30 minutes. The sapphire E_g_ modes at 580.6 and 752.9 cm^−1^ are clearly seen for all samples and the Raman scattering peaks at 1370 cm^−1^ corresponding to the E_2g_ in-plane vibration mode of h-BN^34,35^ are observed from the as-grown and the post-annealed h-BN films at 1500 and 1600 °C as shown in Fig. 1a. Note that the full width at half maximum (FWHM) of the h-BN E_2g_ mode is reduced from 46.33 cm^−1^ for the as-grown h-BN film to 32.96 and 28.84 cm^−1^ after the post-annealing at 1500 and 1600 °C, respectively, as shown in Fig. 1b while the other peaks show negligible change with annealing temperature. A sharper Raman peak at the h-BN E_2g_ mode implies an improvement in the crystallinity of the h-BN film after the post-annealing in N_2_ ambient. The h-BN E_2g_ mode, however, disappears after annealing at 1700 °C, which may be attributed to too high thermal energy that damages the B-N bonds in the h-BN film rather than enhances the crystallinity. Meanwhile, after the post-annealing in a mixture of N_2_ and ammonia (NH_3_), which is used for the nitrogen source in the MOCVD h-BN growth, the E_2g_ mode becomes weak and disappeared as the annealing temperature increases while Raman scattering of A_1_, E_1_, and E_2_ modes of aluminum nitride (AlN) appear and increase due to the nitration of the sapphire (Al_2_O_3_) substrate as shown in Supplementary Fig. S1.

The effects of the post-annealing at 1600 °C in N_2_ ambient, which resulted in much enhanced Raman spectrum, on the structural properties of the h-BN film were further investigated by X-ray reflectivity (XRR), atomic force microscopy (AFM) and TEM. The thickness of the MOCVD-grown h-BN film before (3.078 nm) and after the post-annealing (3.072 nm), estimated by XRR, shows a negligible change while the root mean square roughness obtained by AFM is decreased from 0.202 to 0.163 nm, as shown in Supplementary Fig. S2, indicating a reconstruction of the h-BN film during the high temperature post-annealing process.

**Figure 2:**
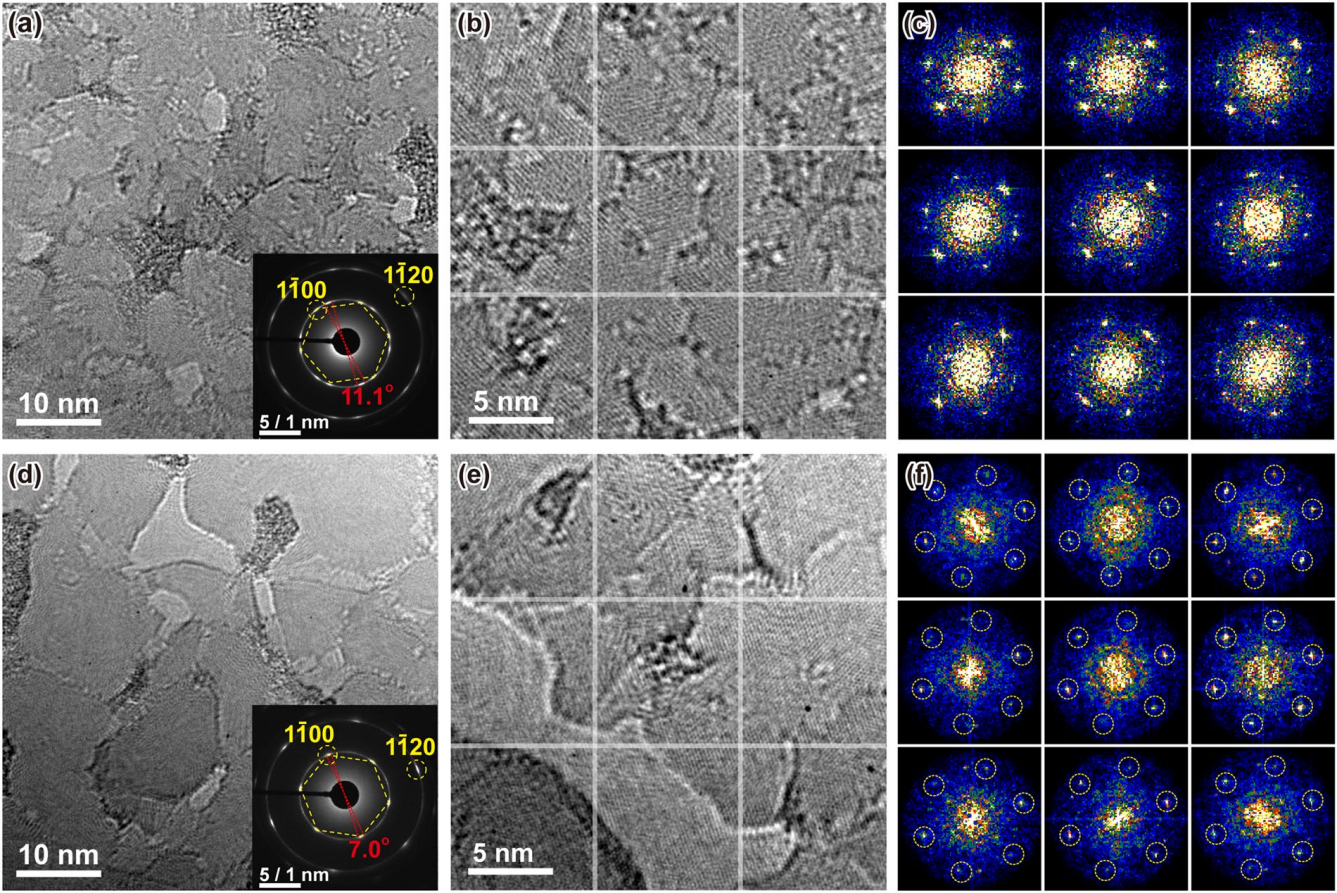
(**a**,**d**) In-plane BF-TEM micrographs at magnification of 200 k×, (**b**,**e**) at magnification of 400 k× and (**c**,**f**) corresponding nine fast Fourier transform (FFT) images in selected areas divided by a 3 × 3 grids indicated in (**b**,**e)** with white solid lines. In (**c)** each FFT pattern shows a slightly-different crystallographic orientation, implying that the as-grown h-BN has a polycrystalline behavior with a domain rotation. The dotted circles in (**f)** indicate that $\{1\bar{1}00\}$ planes and the single domain orientation maintains in the whole FFT pattern in the case of the post-annealed h-BN. Images in upper row are from the as-grown h-BN and those in bottom row are from the post-annealed h-BN at 1600 °C in N_2_ ambient.

Microstructural difference between the as-grown and the post-annealed h-BN films were further investigated by TEM analyses. Low acceleration voltage of 60 kV was used to minimize beam damage on the h-BN samples. Figures 2a,d are the bright field (BF) TEM images and corresponding diffraction patterns of the as-grown and the post-annealed h-BN films under the same magnification and camera length conditions. The diffraction patterns of the as-grown h-BN film consist of two rings corresponding to $\{1\bar{1}00\}$ and $\{11\bar{2}0\}$ planes, indicating that the polycrystalline h-BN film consists of many domains with large variation in crystallographic directions before the annealing, but two crystallographic domains predominantly exist with ~11.1° rotation (inset of Fig. 2a). The diffraction pattern of the annealed h-BN film (inset of Fig. 2d) shows a single six-fold symmetry with less diffused spots having ~7.0° rotation as a result of similar crystallographic directions of the domains constituting the h-BN film. Further investigations of the higher homogeneity and improved crystallinity in the annealed h-BN were performed by higher resolution BF-TEM (Fig. 2b,e) followed by fast Fourier transform (FFT) in the selected area divided by a 3 × 3 grid indicated by white lines in Fig. 2b,e. Corresponding FFT pattern from each grid is placed in the respective position shown in Fig. 2c,f. It is noticeable that nine FFT patterns with a six-fold symmetry, indicated by circles, are coincident in the observed area of 25 × 25 nm^2^ of the annealed h-BN film while the diffraction patterns are varied and rotated along ring-like patterns indicating grain misalignment (polycrystalline) in the as-grown h-BN film. Although variation in contrast in the BF images is observed even in the annealed sample, which is mainly attributed to the thickness variation in the few-layer h-BN film^36^, the FFT patterns in Fig. 2f show that crystallographic homogeneity is remarkably improved, and accordingly the domains are also coarsened by the post-annealing as shown in Fig. 2d. Therefore, it can be inferred that the high temperature annealing contributes to the enhancement in h-BN crystallinity, which also corresponds to the sharper E_2g_ Raman peak in the post-annealed h-BN.

**Figure 3:**
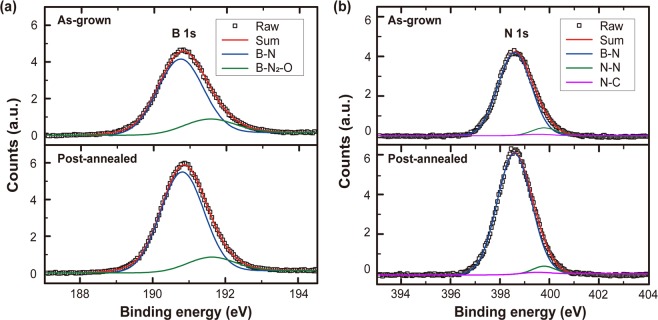
XPS deconvolution results of (**a**) B 1s and (**b**) N 1s regions obtained from the as-grown h-BN film and the post-annealed h-BN film.

Figure 3a,b are B 1s and N 1s core-level spectra measured from the as-grown and the post-annealed h-BN films by X-ray photoelectron spectroscopy (XPS), respectively. The FWHM of the B 1s and the N 1s peaks becomes lower by 0.112 and 0.029 eV, respectively, after the post-annealing at 1600 °C in N_2_ ambient as shown in Table 1. According to H. Sediri *et al*.^37^, not only B-N bonding in *sp*^2^-BN film but also N-N or B-N_2_-O bonding, which is attributed to intrinsic stable defects such as self-interstitial N_i_, nitrogen vacancy and substituted oxygen atom in nitrogen site, can contribute to B 1s and N 1s core-level XPS spectra. The XPS peaks were deconvoluted by using Gaussian-Lorentzian fitting to extract each contribution to the whole spectrum. The main peaks at the binding energy of 190.8 eV for the B 1s and 398.6 eV for the N 1s are in good agreement with the reported values of B-N bonding^20,38^. In addition, shifted peaks toward higher binding energy by 0.8 eV than the B-N bonding at 191.6 eV in the B 1s and 398.8 eV in the N 1s spectra are attributed to B-N_2_-O and O-B-N or N-N bonding, respectively, because of electronegativity difference between oxygen and nitrogen^37,39^. There is no noticeable change in the position of peaks but relative ratio of defective bonding to the B-N bonding is reduced in the post-annealed h-BN film (Table 1), indicating an enhanced crystallinity of the h-BN film by the post-annealing.

**Table 1:** FWHM of B 1s and N 1s XPS core level spectra and relative ratio of deconvoluted bondings to B-N bonding.

	B 1s FWHM (eV)	N 1s FWHM (eV)	B-N_2_-O/ B-N ratio	N-N/ B-N ratio
As-grown	1.678	1.710	0.206	0.070
Post-annealed	1.566	1.681	0.150	0.041

**Figure 4:**
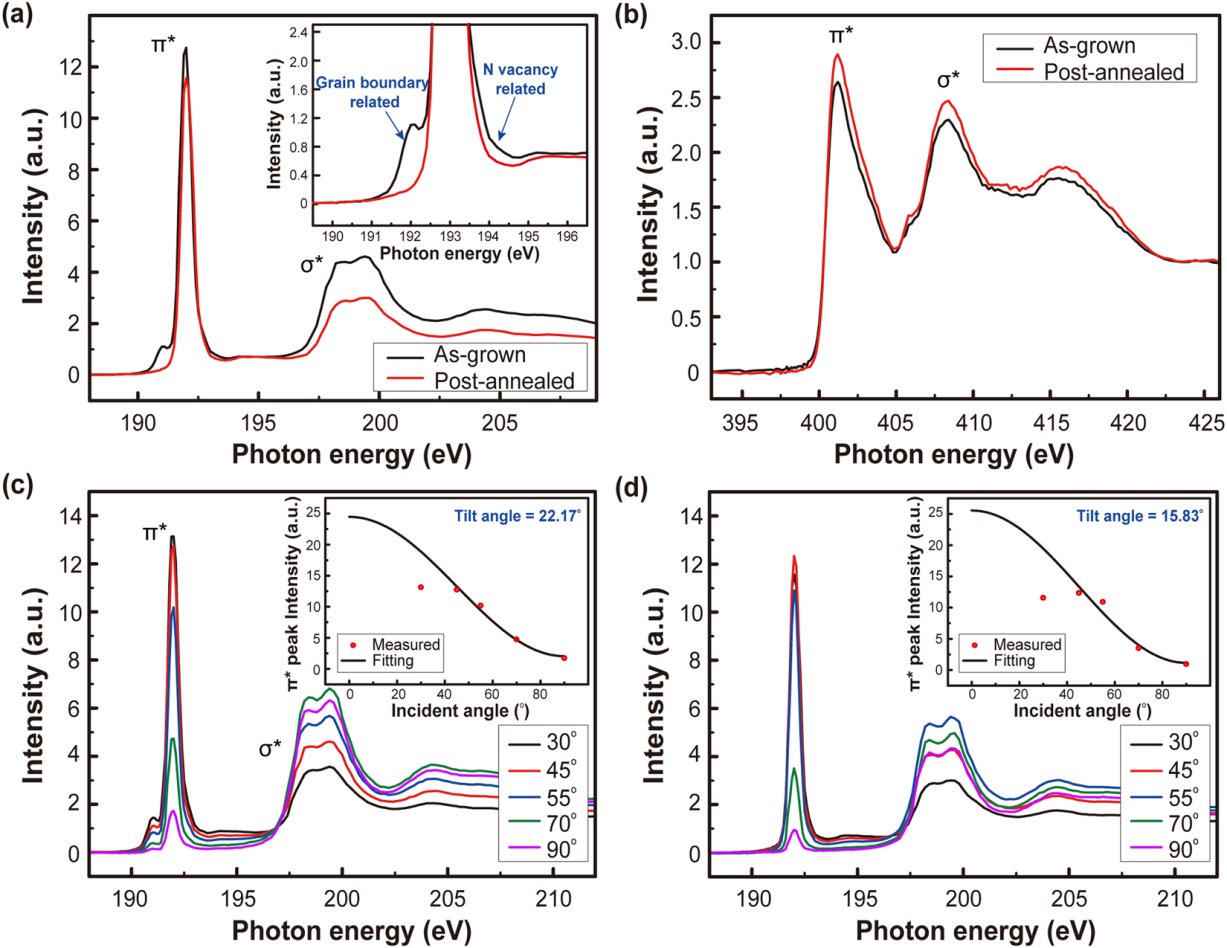
Comparison of NEXAFS (**a**) B K-edge and (**b**) N K-edge spectra of the h-BN films before and after the post-annealing. NEXAFS was measured at X-ray incident angle of 30° for the h-BN films. NEXAFS B K-edge spectra of (**c**) the as-grown and (**d**) the post-annealed h-BN films with different X-ray incident angles of 30, 45, 55, 70, and 90° are shown. Inset shows intensity of π* peak in NEXAFS B K-edge spectra as a function of X-ray incident angle (red open circle) and the calculated intensity (black solid line) for estimation of the average tilt angle.

Further investigation on the effect of the high temperature annealing on the microstructure of the h-BN film was performed by NEXAFS measurement, which is a useful tool to obtain information on the local geometric and electronic structure^40,41^, at B K-edge and N K-edge with X-ray incident angle of 30°. Both samples show the peaks at ~192.0 eV in B K-edge (Fig. 4a) and ~401.2 eV in N K-edge spectra (Fig. 4b) ascribed to the transition from B 1s and N 1s core-levels to the π* anti-bonding orbital states of the *sp*^2^-BN film while the double peak at ~198 eV in B K-edge and the single peak at ~408 eV in N K-edge spectra are contributed by the transitions from the core levels to σ* states. Interestingly, noticeable change in the shoulders of the π* peak in the B K-edge spectra is observed as shown in inset of Fig. 4a. The shoulder peaks in B K-edge NEXAFS spectra are related to atomic defects and disorders in h-BN films. The shoulder in the lower photon energy side represents the existence of the boron atoms bonded to the four neighbor nitrogen atoms (B-N_4_)^42^ while the higher photon energy shoulder is attributed to nitrogen vacancies and an incomplete phonon relaxation^43^. Therefore, the decrease in both shoulders near the π* peak in the B K-edge spectra can be interpreted as a reduction of structural imperfections including grain boundaries and nitrogen vacancies by the post-annealing. NEXAFS B K-edge and N K-edge spectra were also measured for the post-annealed h-BN film under mixture of N_2_ and NH_3_ ambient as shown in Supplementary Fig. S3. Absorption transitions from 1s to only σ* anti-bonding states^44,45^ are observed instead of the transition from 1s to π* anti-bonding states after the annealing, in agreement with the result of Raman spectra.

In addition, NEXAFS spectra as function of X-ray incident angle, defined as the angle between the polarization angle of the incident X-ray beam and the surface normal to the h-BN films, were measured in order to estimate the average tilt angle of the h-BN films as shown in Fig. 4 by using the following equation^26,46^:

1 $$I=C\cdot \frac{P}{3}\,\{1+\frac{1}{2}(3{\cos }^{2}\theta -1)(3{\cos }^{2}\alpha -1)\}+\frac{(1-P)}{2}{\sin }^{2}\alpha $$

where, *I* is the intensity of X-ray absorption due to transition from 1s core-level to π* anti-bonding states, *C* is a constant, *P* is the degree of polarization, which is determined to be 0.85 by the experimental equipment, $\theta$ is the polarization angle of the incident X-ray beam, and *α* is the average tilt angle. The average tilt angle quantifies that how much the *sp*^2^-bonded layer material is aligned parallel to the surface, and it is strongly depends on the change in intensity of the transition from 1s core level to π* anti-bonding states at different X-ray incident angles. In the case of perfectly aligned layered materials parallel to the surface, the average tilt angle is 0° as a result of no observed π* peak at the X-ray incident angle of 90°, and it increases as the amount of structural imperfection increases^25,26^. Figure 4c,d are the angle-dependent NEXAFS B K-edge spectra for the as-grown and the post-annealed h-BN films, respectively. The intensity of π* peak decreases as the X-ray incident angle increases from 30 to 90° for both samples, but its decreasing ratio is different. The average tilt angle, obtained by fitting the measured data using the Eq. 1, for the post-annealed h-BN is 15.83°, much lower than that for the as-grown h-BN (22.17°), indicating the improvement of the quality of the layered h-BN films as a result of the highly ordered domains after the post-annealing, which is consistent with Raman, TEM and XPS results.

Effects of thermal annealing on the structural properties of graphene have been reported by computational studies^47,48^ such as Monte Carlo simulation. According to M. Becton *et al*.^47^, individual atoms with increased kinetic energy are rearranged during the high temperature annealing process, and subsequently imperfection of crystal such as unstable few-membered rings can be eliminated to be a more stable state. Coalescence of domains or gradual change in the orientation of the domains as the effect of annealing process has been suggested by J. Zhuang *et al*.^48^ as well. Analogous to thermally annealed graphene layers, remarkably enhanced structural properties of the MOCVD-grown h-BN films, observed by Raman spectroscopy, TEM, AFM, XPS, and NEXAFS, after the high temperature post-annealing are attributed to the reconstruction of the MOCVD-grown defective h-BN layer initiated by the mobile atoms with sufficient kinetic energy during the annealing process.

**Figure 5:**
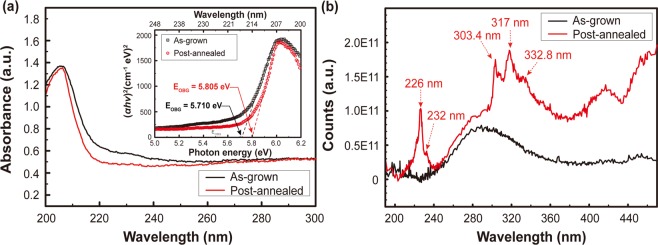
(**a**) UV-visible absorbance spectra measure at 10 K and corresponding Tauc’s plot for optical band gap of direct transition estimation for the as-grown and the post-annealed h-BN films is shown in inset. (**b**) Comparison of PL spectra of the h-BN films measured at 10 K as well on the sapphire substrate before and after the post-annealing.

Improved structural properties as a result of the high temperature post-annealing can affect the optical properties of the h-BN film. Figure 5a shows the absorbance spectra measured at 10 K and estimated optical bandgap from the Tauc’s plot (inset) of the h-BN film before and after the post-annealing. Low temperature absorption measurement gives advantages on obtaining larger excitonic transition signal and preventing the intrinsic transition from being suffered by the impurity-related ones, which can be activated at room temperature^49^. Both samples show abrupt increase in absorbance near 220 nm and peaks at ~205 nm with similar intensity as shown in Fig. 5a. One noticeable feature is the long-wavelength absorption tail near the absorption edge that is remarkably reduced for the post-annealed h-BN film, which reflects the suppressed broadening of states caused by structural disorder^50^. The optical band gap estimated from corresponding Tauc’s plot is enlarged from 5.710 eV to 5.805 eV due to less distributed states below the absorption edge extending into the gap as a result of the improved crystallinity of the post-annealed h-BN film.

In addition, Photoluminescence (PL) spectra of the h-BN films were measured under the excitation wavelength of 170 nm at 10 K as shown in Fig. 5b. Note that cryogenic PL measurements performed to resolve fine structures of excitonic states by preventing thermal dissociation and thus clearly observe the post-annealing effect on the microstructural properties of h-BN films. Excitonic peaks comparable to commercial h-BN pristine powder (Graphene Supermarket, Supplementary Fig. S4) are observed from the post-annealed h-BN film while no exciton-related peak is observed from the as-grown h-BN film that is attributed to the disordered h-BN structure quenching the excitonic PL^51–53^. The transition peaks related to a donor-acceptor pair (DAP) transition at 303.4 nm together with its replicas at 317 and 332.8 nm^54^, as well as the emission peaks at 226 and 232 nm originating from the excitons localized at certain structural defects^55^ appear after the post-annealing. Although near band-edge phonon replicas of indirect exciton resulting in emission at wavelength shorter than 215 nm is not observed, we believe that remarkable enhanced PL enabled by improved crystallinity after the post-annealing can provide a great opportunity for use of the large-area, multi-wafer scale h-BN film grown by MOCVD as a highly efficient active material in optoelectronic applications.

## Conclusions

In conclusion, h-BN films were grown on 2-inch sapphire substrates at moderate growth temperature (1050 °C) by the conventional multi-wafer MOCVD system, followed by the post-growth annealing at high temperature over 1500 °C in order to improve the structural and optical properties of the h-BN films. The effects of the post-annealing on the structural properties of h-BN films were investigated by various spectroscopic and microscopic techniques. Sharper Raman scattering of in-plane E_2g_ mode and B-N 1s core levels in the XPS spectra, significantly reduced defect-related shoulder peaks in NEXAFS, and higher crystallographic homogeneity by TEM analysis were observed after the post-annealing of the h-BN film at 1600 °C in comparison with those from the as-grown h-BN film. It was found that the enhanced microstructural properties by the post-annealing directly results in an abrupt increase in PL by excitonic transitions, as well as enlarged optical band gap in the h-BN. We believe that the post-annealing of the h-BN film can provide a delightful way to obtain highly crystalline and multiple wafer-scale MOCVD-grown h-BN films for their practical applications in 2D electronic and optoelectronic devices operating in DUV ranges.

## Methods

### Growth of h-BN film by using MOCVD

h-BN films were grown on 2-inch double side polished sapphire substrates by using a commercial MOCVD system at 1050 °C under reactor pressure of 30 mbar with H_2_ carrier gas. A pulsed source-injection mode^25,26^ composed of 4 steps in a single pulse cycle; injection of 10 sccm triethylborane (TEB) for 4 seconds, interruption for 2 seconds, injection of 8,000 sccm NH_3_ for 4 seconds, and interruption for 2 sections was used. Total pulse cycles of 200 resulted in the growth of 9 ~10 layers of h-BN.

### Post-annealing of the h-BN film

The MOCVD-grown h-BN films were thermally annealed in N_2_ or a mixture gas of NH_3_ and N_2_ at 1500 ~1700 °C for 30 min. The pressure of N_2_ was 95 kPa, and the gas flow rate ratio of a mixture gas was NH_3_:N_2_ = 5:27. The surface of the h-BN film was covered by a sapphire wafer in order to suppress the decomposition of h-BN during the annealing process.

### Characterizations

For the spectroscopic characterization, Raman spectroscopy was performed for the as-prepared samples without transfer process by using a WITec micro-Raman spectrometer system under 532 nm Nd:YAG laser excitation.

XRR measurements for thickness estimation of the MOCVD-grown h-BN film on sapphire substrate were carried out by using an X-ray diffractometer (PANalytical) with Cu-Kα (λ = 1.5406 Å) radiation. The measured XRR data were analyzed by using GenX software.

Surface morphology of the h-BN films were observed by Veeco Dimension 3100 AFM with a tapping mode. TEM analysis was carried by using a JEOL ARM200F TEM operated at 60 kV. In-plane high-resolution TEM images were able to be obtained by the transfer of the h-BN film onto the TEM grid by the poly(methyl methacrylate) (PMMA)-assisted method, described in the following section.

XPS and NEXAFS spectrum were measured at 4D beam line in Pohang Accelerator Laboratory, South Korea in order to investigate the chemical bonding states of the MOCVD-grown and post-annealed h-BN films, which were transferred onto the conductive silicon substrate by using the PMMA-assisted method as well. The B 1s and N 1s core-level XPS were measured using a synchrotron radiation photoemission spectroscopy with the incident X-ray source at 350 and 550 eV, respectively. The NEXAFS spectra were obtained with total electron yield detection at various angles of incidence of the synchrotron photon beam with respect to the surface normal of the specimen.

Optical characterization of the h-BN films were carried by using the synchrotron radiation at the BL03A high flux VUV beam line at National Synchrotron Radiation Research Center, Taiwan at 10 K. The details of the experimental end-station for measurement of absorption and photoluminescence spectra were reported by H.-C. Lu *et al*.^56^. In this work, a monochromator (iHR 320, HORIBA) equipped with a diffraction grating of 1800 lines/mm and slit size of 1 × 1 mm^2^ and a photomultiplier tube (PMT, Hamamatsu R943–02) were used for the PL measurement. The entrance and exit slit widths were set at 300 and 5,000 μm, respectively.

### Transfer method

After spin coating of PMMA on the h-BN film on sapphire, and the PMMA-coated h-BN sample was immersed into diluted hydrofluoric acid solution (HF:H_2_O = 1:9) at room temperature. After few seconds, the h-BN film completely delaminated from the sapphire substrate. Floating PMMA/h-BN film was moved onto the distilled water for cleaning, and finally it was fished by a target substrate such as silicon substrate. The transferred film was allowed to dry in air and then immersed into acetone to remove the PMMA on the h-BN film. Vacuum annealing at temperature of approximately 320 °C and pressure below 5 × 10^−6^ Torr was performed right before the characterization in order to remove residual hydrocarbon on the surface.

## Supplementary information

Supplementary information

## Data Availability

The datasets generated and/or analyzed during the current study are available from the corresponding author on reasonable request.
