# Supplementary material for: Improvements in structural and optical properties of wafer-scale hexagonal boron nitride film by post-growth annealing
Source: Sci Rep. 2019 Jul 22;9:10590. doi: 10.1038/s41598-019-47093-9 (PMC6646322; doi:10.1038/s41598-019-47093-9)
Supplement: Supplementary file 1 — Supplementary information [file 41598_2019_47093_MOESM1_ESM.pdf]

Supplementary Information

Improvements in structural and optical properties of wafer-scale hexagonal boron nitride film by post-growth annealing

Seung Hee Lee[1], Hokyeong Jeong[1], Odongo Francis Ngome Okello[1], Shiyu Xiao[2], Seokho Moon[1], Dong Yeong Kim[1], Gi-Yeop Kim[1], Jen-Iu Lo[3], Yu-Chain Peng[3], Bing-Ming Cheng[3], Hideto Miyake[2], Si-Young Choi[1], and Jong Kyu Kim[1]*

[1] Department of Materials Science and Engineering, Pohang University of Science and Technology (POSTECH), Pohang 37673, Republic of Korea

[2] Graduate School of Regional Innovation Studies, Mie University, Tsu 514-8507, Japan

[3] National Synchrotron Radiation Research Center, Hsinchu 30076, Taiwan

1. Raman spectra of the post-annealed MOCVD-grown h-BN films in mixture of NH_3 and N_2 ambient

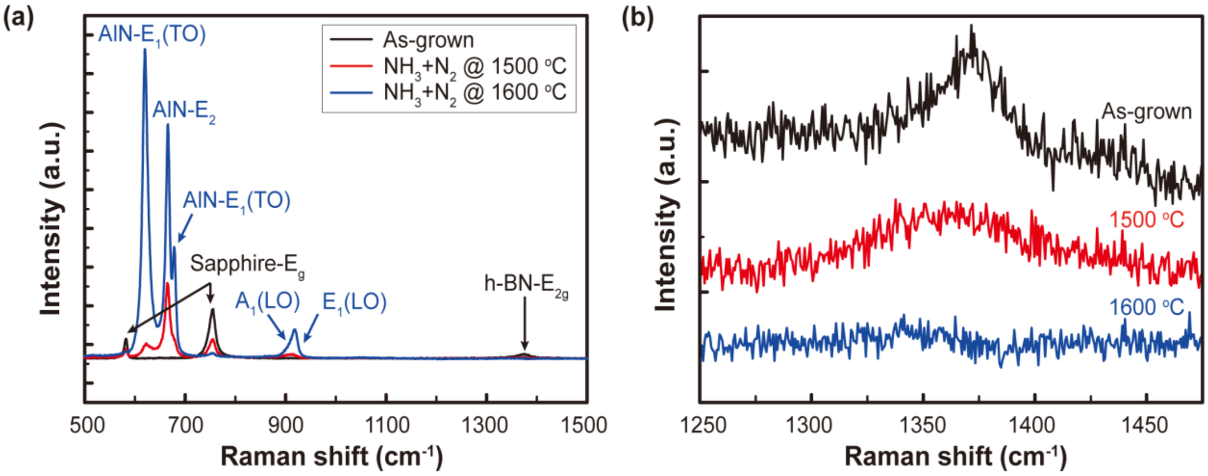

Supplementary Figure S1. (**a**) Comparison of the full Raman spectra of the MOCVD-grown h-BN film on sapphire before and after the post-annealing at from 1500 to 1600 °C in mixture gas of NH_3 and N_2 ambient for 10 min. (**b**) Magnified Raman spectra of the h-BN E_{2g} peaks.

Supplementary Fig. S1 shows Raman spectra of the as-grown and post-annealed MOCVD-grown h-BN films on sapphire substrate at different annealing temperature in mixture gas of NH_3 and N_2 ambient. As shown in Supplementary Fig. S1a, full Raman spectra from 500 to 1500 cm^{-1}, the sapphire E_g phonon modes at 580.6 and 752.9 cm^{-1} decrease while Raman-active modes of aluminum nitride (AlN) are enhanced, i.e., Raman peaks of A_1 transverse optical (TO), E_2, E_1(TO), A_1 longitudinal optical (LO), and E_1(LO) modes at 612, 655, 668, 891, and 907 cm^{-1}, respectively[1,2], increase as increase the annealing temperature from 1500 to 1600 °C due to nitridation of the Al_2O_3 to AlN. Meanwhile, Raman scattering peaks at 1370 cm^{-1} corresponding to the E_{2g} in-plane vibration mode of h-BN decrease with much larger FWHM of 80.02 cm^{-1} than the as-grown h-BN (46.33 cm^{-1}) after the post-annealing at 1500 °C. Moreover, the post-annealed h-BN film at 1600 °C under mixture gases of NH_3 and N_2 shows disappeared in-plane vibration mode of the h-BN indicating damaged the *sp*^{2} B-

N bonds owing to the formation of AlN.

2. Thickness and surface morphology of the post-annealed MOCVD-grown h-BN films

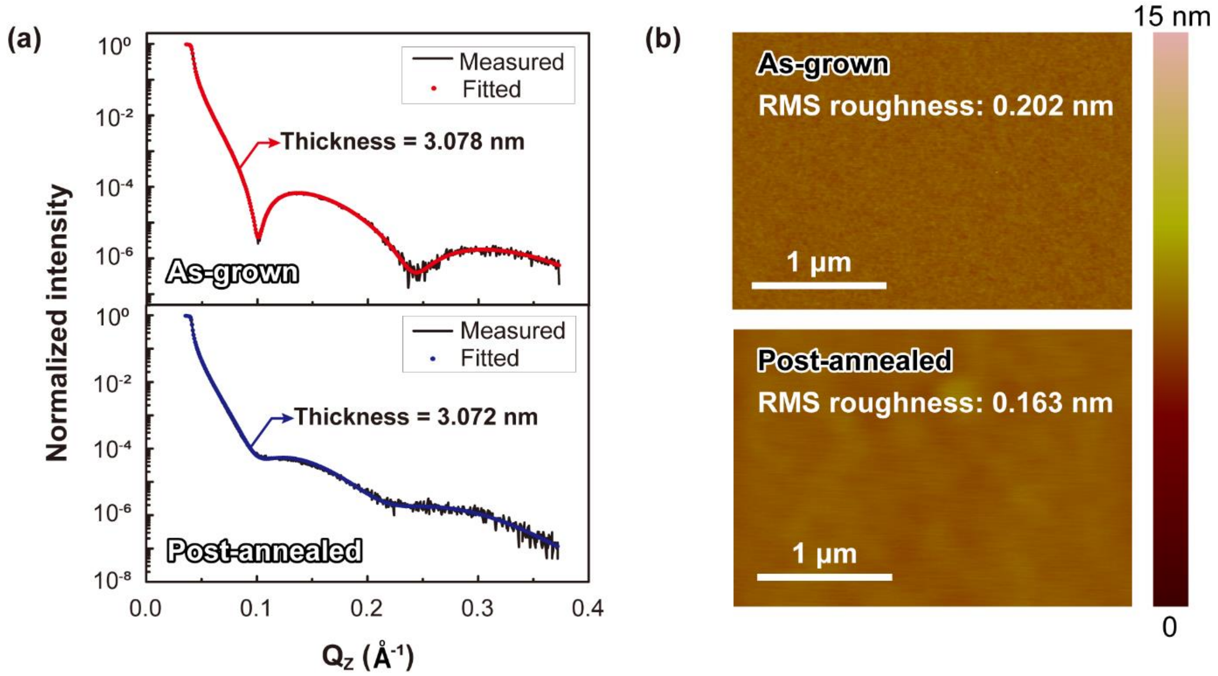

Supplementary Figure S2. Comparison of the thickness and surface morphology obtained by (**a**) X-ray reflectance (XRR) and (**b**) atomic force microscopy (AFM), respectively, of the MOCVD-grown h-BN film on sapphire and that after the post-annealing at 1600 °C.

The h-BN film thickness was deduced by fitting the corrected X-ray reflectance (XRR) data, which is the normalized reflectance considering geometric factor of the sample size as function of the scattering vector of the X-ray along the surface normal direction, Q_z. According to the Fresnel reflection, the reflectivity of nth layers is calculated by the equation as below:

$$R_n = \frac{r_{n,n+1} + R_{n+1}\exp[2id_{n+1}k_{z,n+1}]}{1 + r_{n,n+1}R_{n+1}\exp[2id_{n+1}k_{z,n+1}]}$$

where d_n is the thickness of nth layer, and $r_{n,n+1}$ is the Fresnel coefficient at n^{th}

interface, $r_{n,n+1} = \frac{k_{z,n}-k_{z,n+1}}{k_{z,n}+k_{z,n+1}} \exp[-2\sigma_{n+1}^2 k_{z,n} k_{z,n+1}]$, of which σ is a parameter of the surface roughness.

GenX, which is a refinement program calculating the optical reflectivity of X-ray from flat surface based in Parratt's recursion formula, was used to optimize the parameter of each layer corresponding to the experimental XRR spectrum data[3,4].

3. NEXAFS of the post-annealed MOCVD-grown h-BN films in mixture of NH_3 and N_2 ambient

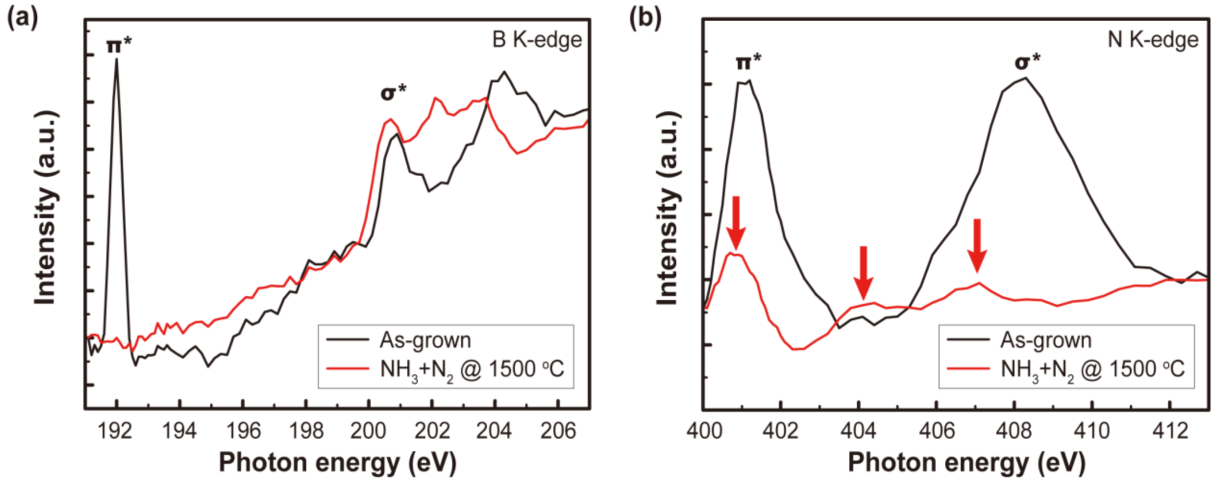

Supplementary Figure S3. Comparison of the NEXAFS TEY spectra of the MOCVD-grown h-BN film on sapphire before and after the post-annealing at 1500 °C in mixture gas of NH_3 and N_2 ambient (**a**) at B K-edge and (**b**) N K-edge measured at X-ray incident angle of 45° for the h-BN films.

Further investigation of the electronic structure and local geometry of the post-annealed h-BN film is revealed by NEXAFS experiments. Although conductive substrate is preferred for the NEXAFS measurement to prevent the charging effect, total electron yield (TEY) intensity was obtained from the h-BN film on the insulating sapphire substrate mounted with a conductive bridge because the post-annealed h-BN films on the nitrided substrate was

not delaminated from the substrate by using hydrofluoric acid (HF), which is an etchant for Al_2O_3 and a delaminating solution for the h-BN film grown on sapphire in the transfer process. In spite of low TEY intensity as a result of the charging effect, there are apparent changes in the π* and σ* peaks of the h-BN films as shown in Supplementary Fig. S3. Supplementary Fig. S3a shows NEXAFS TEY spectra at B K-edge measured from the as-grown and the post-annealed h-BN film at 1500 °C in the mixture of NH_3 and N_2 ambient. The peaks of π* and σ* transitions are clearly distinguished in NEXAFS of the as-grown h-BN film around the B K-edge, while only σ* transitions are present in NEXAFS of the post-annealed h-BN film indicating the destruction of sp^2-hybridized boron. The transitions from N 1s to π* and σ* states are clearly observed as well in the as-grown h-BN film as shown in Supplementary Fig. S3b. After the post-annealing, however, three peaks at around 401, 404, and 407 eV (red arrows in Supplementary Fig. S2b) that correspond to a combination of π- and σ-bond characters of the wurtzite structure of AlN appear in N K-edge NEXAFS spectra[5,6].

4. Photoluminescence of the h-BN

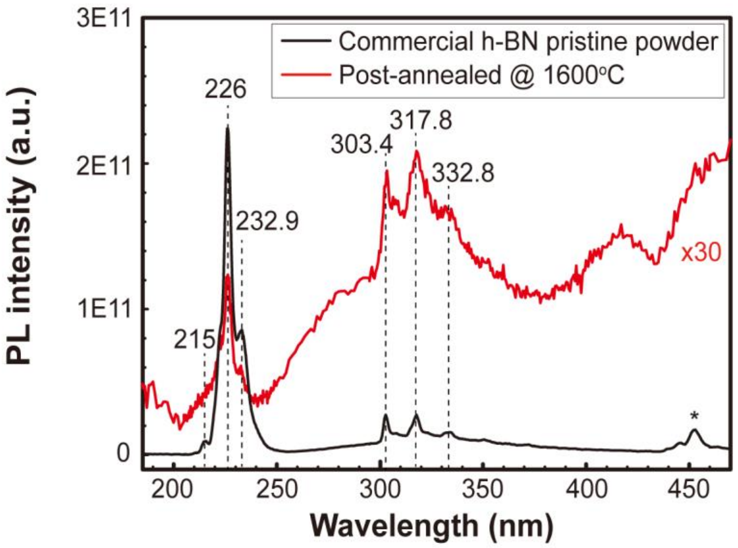

Supplementary Figure S4. Photoluminescence spectra measured under excitation with

synchrotron radiation of 170 nm at 10 K from the commercial h-BN pristine powder (black) and the post-annealed h-BN film (red) at 1600 °C in N_2 ambient for 30 min whose intensity is multiplied by 30. The star indicates the second order of the 226 nm band.

Comparison of the photoluminescence (PL) spectra from the commercial h-BN pristine powder (supplied from Graphene Supermarket) and the h-BN film after the post-annealing at 1600 °C in N_2 ambient is shown in Supplementary Fig. S4. A near band-edge phonon replica of indirect exciton resulting in emission at wavelength around 215 nm is observed from the commercial h-BN pristine powder while the luminescence of the 226 nm and 232 nm bands attributed to bound exciton luminescence from the structural defects is dominant due to easily pressed and deformed powder h-BN[7] in the sample holder. The transition peaks related to a donor-acceptor pair (DAP) transition at 303.4 nm together with its replicas at 317 and 332.8 nm are clearly shown in the PL spectra of the h-BN pristine powder as well. The PL peaks from the post-annealed h-BN film are in good agreement with that from the commercial h-BN pristine powder, which consists of single crystals, except absent of the near band-edge peak at around 215 nm.

References

1. Wu, Q. *et al*. Extented Vapor-Liquid-Solid Growth and Field Emission Properties of Aluminum Nitride Nanowires. *J. Mater. Chem*. **13,** 2024-2027 (2003).

2. Lei, W. *et al*. Three-Dimensional AlN Microroses and Their Enhanced Photoluminescence Properties *Chem. Commun*. **41,** 5221-5223 (2008).

3. Yasaka, M. X-ray Thin-Film Measurement Techniques: V. X-Ray Reflectivity Measurement. *Rigaku J.* **26,** 1-9 (2010).

4. Bjorck, M. & Andersson, G. GenX: An Extensible X-Ray Reflectivity Refinement Program Utilizing Differential Evolution. *J. Appl. Cryst.* **40,** 1174-1178 (2007).

5. Lawniczak-Jablonska, K. *et al*. Electronic States in Valence and Conduction Bands of Group-III Nitrides: Experiment and Theory. *Phys. Rev. B* **61,** 16623 (2000).

6. Pao, C. W. *et al*. Electronic Structures of Group-III-Nitride Nanorods Studied by X-Ray Absorption, X-Ray Emission, and Raman Spectroscopy. *Appl. Phys. Lett.* **88,** 223113 (2006).

7. Watanabe, K., Taniguchi, T., Kuroda, T. & Kanda, H. Effects of Deformation on Band-Edge Luminescence of Hexagonal Boron Nitride Single Crystals. *Appl. Phys. Lett.* **89,** 141902 (2006).
